# Amino acid storage: lysosomal double role in health and disease

**DOI:** 10.1080/27694127.2025.2498324

**Published:** 2025-05-08

**Authors:** Aiswarya Raj, Samantha Shrihari, Urmi Bandyopadhyay

**Affiliations:** a Manipal Institute of Regenerative Medicine (MIRM), Bengaluru, India; b Manipal Academy of Higher Education (MAHE), Manipal, Karnataka, India

**Keywords:** Lysosome, nutrient storage, leucine, LAMTOR-RAG GTPase complex, Extracellular Environment

## Abstract

Cellular homeostasis depends on a multitude of cellular functions, which in turn depend on the clearance of damaged components for their maintenance. Lysosomes being one of the main sites of recycling, are at the frontline for cellular protein degradation, which leads to generation of protein building blocks, the amino acids (AAs), within the lysosomal lumen. However, the fate of these lysosomal pool of AAs are only partly known. Recently, studies from our and other groups have led to the finding that AA can be stored in lysosomes and revealed a homeostatic communication of these storages with the environment. Thus, lysosome appear to be a nutritional signaling hub that has a dual role. As a degradation-competent hydrolytic sack, lysosomes have a long-studied degradative function, additionally now they can either store or channel into utilization of the AAs generated through their proteolytic activity. Since the existence of a lysosomal AA storage pool has been determined by changing the levels of extracellular AAs, this indicates a multi-directional homeostatic communication between the lysosome and the extracellular environment. This Lysosomal homeostatic and adaptive response to the niche could be vital for life-threatening age-related degenerative disorders, where the lysosome-autophagy pathway and the microenvironmental cues play major roles in the disease progression, which will be discussed further in this piece.

The regulation of AA-homeostasis by mTORC1 (mechanistic target of rapamycin complex-1) pathway has been extensively investigated. However, the cellular AA mobilization, storage in the cell, and utilization of the storages remain understudied. Furthermore, how the intracellular AA storages respond to alterations in the microenvironment remains unknown.

By tracking AA movements in and out of the cells, using radioactive AA tracing, we uncovered a unique role of lysosomes in AA storage and homeostasis in response to the changes in extracellular AA composition. In this context, a fraction of the lysosomal essential AAs is exported to the extracellular environment when present in the extracellular milieu. However, this export is entirely and rapidly blocked upon deprivation of the same AAs in the extracellular milieu, leading to the generation of an AA pool stored within lysosomes. This phenomenon has been best described and most prominent for the most abundant essential AA, leucine. Further, the generated lysosomal leucine reservoir is utilized for cellular protein synthesis. Interestingly, AA homeostasis is mostly regulated by mTORC1 kinase, we showed that this novel leucine-storage pathway is mTORC1 kinase-independent. Intriguingly, our research rather revealed the involvement of some other key members of the canonical mTORC1 signaling pathway, in particular, the LAMTOR-RAG GTPase complex. To the best of our knowledge, this is the first report of a mTORC1-independent function of LAMTOR-RAG GTPase complex, presumably to sustain cellular growth and survival in an austere environment [1].

The balance between AA storage and export could be important for supporting cell survival or growth within a cell population, as well as for tissue physiology and homeostasis at the organismal level. These exported nutrients can be shared by neighboring cells, but their impact on them and the mechanism of sharing require further investigation. Along with other functions the cell autonomous growth advantage is potentially gained by funneling the lysosomally stored AA pool for cellular protein synthesis. On the other hand, hypothetically, as leucine starvation generates cellular leucine storage that can trigger cellular AA toxicity, which can be handled well by secluding cellular AA overload within single membrane-bound lysosomes.

It can be speculated that during lysosomal AA storage pool generation, mobilization of AA from one cellular location and compartment to the other would be necessary. This could potentially be regulated by modulating the opening and closing of yet to be identified channels, transporters and/or permeases on lysosomal and/or cellular membranes, which requires future attention. Extracellular AA levels sensing through those sensors, channels and transporters may influence lysosomes to decide whether to store, export, and/or shunt stored AAs for protein translation or for other functions, for better adaptation to the surroundings.

During our research, a series of pharmacological/genetic studies has sparked the idea of the existence of novel complexes distinct from the conventional mTORC1 complex on the lysosomal membrane regulating the lysosomal AA storage, including RagGTPases: especially RagGTPaseA and RagGTPaseB and Ragulators: especially Lamtor1 and Lamtor2, which are functioning in mTORC1 kinase-independent manner. Moreover, how the mTORC1 complex regulates AA import-export independently of mTORC1 kinase also needs additional investigations. Other kinase-independent functions of the mTORC1 signaling cascade have already been identified. For example, the transcription factor TFEB and the AA transporter SLC38A9 also bind to Rag GTPases, but the role of these factors in lysosomal AA storage is unknown. Further, our findings implicate the use of lysosomally-stored AAs in mRNA translation, presumably to support synthesis of yet to be identified selective proteins that are vital for survival under starvation. In addition to protein–protein interactions, protein–lipid interactions could be important future directions to explore in this context.

Although differential lysosomal storage-transport patterns of essential AAs versus non-essential AAs have been shown, however beyond lysosomes, how other organellar crosstalks in coordination with lysosomes dictate the lysosomal AA storage-export processes in physiological and pathological contexts remains to be elucidated.

Furthermore, we observed impairment in lysosomal leucine storage in the MCF7 breast cancer cell line, which may suggest a potential clinical importance of this lysosomal AA storage pathway in cancer [1]. In cancer biology, the nutrient-deficient tumor core could possibly accentuate lysosomal AA storage and further their utilization/usage in cellular protein translation. Therefore, altering AA storage and usage by changing the local nutrient supply, presumably with the help of targeted nano-bioengineering techniques, could be therapeutically effective. As most of the data is collected majorly in MCF7 cell line, requirement of investigation in other cell lines, more importantly in vivo model systems, is undeniable.

Investigation of neurodegeneration, including in the context of lysosomal storage disorders and aging/senescence, in the light of lysosomal-AA storage-homeostasis, is important. In neurodegeneration, dietary-AA supplementation treatment is gaining popularity owing to the increasing success rate and ease of therapeutic regime. It indicates niche AA can control/regulate disease progression. Simultaneously, in neurovegetative disorders cytosolic/lysosomal abnormal protein aggregate formation can potentially affect lysosomal AA composition and lysosomal AA storage-homeostasis. Altogether, pointing toward the importance of niche guided lysosomal-AA storage-homeostasis in neurodegeneration. Deeper research along the line will empower us to tackle degenerative disease progression in a non-invasive manner.

In summary, our findings showed as part of the global cellular metabolism, lysosomes can play an important role. As lysosomes can receive environmental inputs that can trigger either lysosomal AA storage or AA export, which in turn can alter downstream signaling leading to changes at the physiological as well as pathological levels, these aspects require further investigation.

## References

[1] Bandyopadhyay U, Todorova P, Pavlova NN, et al. Leucine retention in lysosomes is regulated by starvation. Proc Natl Acad Sci USA. 2022;119(6). doi: 10.1073/pnas.2114912119 PMC8833167 35105808

